# The Association between Malaria and β-Carotene Levels: A Systematic Review and Meta-Analysis

**DOI:** 10.3390/antiox12091687

**Published:** 2023-08-29

**Authors:** Kwuntida Uthaisar Kotepui, Aongart Mahittikorn, Polrat Wilairatana, Frederick Ramirez Masangkay, Manas Kotepui

**Affiliations:** 1 Medical Technology, School of Allied Health Sciences, Walailak University, Thasala, Nakhon Si Thammarat 80160, Thailand; 2 Department of Protozoology, Faculty of Tropical Medicine, Mahidol University, Bangkok 10400, Thailand; 3 Department of Clinical Tropical Medicine, Faculty of Tropical Medicine, Mahidol University, Bangkok 10400, Thailand; 4 Department of Medical Technology, Faculty of Pharmacy, University of Santo Tomas, Manila 1008, Philippines

**Keywords:** malaria, oxidative stress, β-carotene, vitamin A, carotenoid, systematic review

## Abstract

Background: β-Carotene, which is a prominent carotenoid with notable antioxidant properties, may play a role in countering the oxidative stresses induced by malaria. The association between β-carotene levels and malaria is not yet fully understood, prompting this systematic review and meta-analysis. Methods: A rigorous search of databases, including Nursing and Allied Health Premium, EMBASE, MEDLINE, Ovid, PubMed, Scopus, and Google Scholar, was undertaken to collate studies that focused on β-carotene levels in malaria patients. The selected studies underwent critical appraisal, followed by data extraction for a meta-analysis. Results: Of the 2498 records initially identified, 10 were deemed suitable for synthesis. A considerable number of these studies indicated a pronounced reduction in β-carotene levels among malaria patients in contrast with uninfected individuals. The meta-analysis, encompassing 421 malaria patients and 240 uninfected controls, revealed a significant correlation between reduced β-carotene levels and malaria (*p* < 0.01, Hedges’s g: −1.26, 95% CI: −2.00–(−0.53), I^2^: 93.86%, seven studies). Conclusions: The conducted systematic review and meta-analysis corroborated the correlation between lower β-carotene levels and malaria. The intricate relationship between malaria and β-carotene merits deeper exploration. A comprehensive understanding of this association might pave the way for innovative therapeutic approaches leveraging the antioxidant attributes of β-carotene to combat malaria-induced oxidative stress.

## 1. Introduction

Malaria is a parasitic disease primarily caused by the *Plasmodium* species and is transmitted to humans through bites from infected *Anopheles* mosquitoes [1]. Five *Plasmodium* species were identified as causative agents of malaria in humans: *P. falciparum*, *P. vivax, P. ovale*, *P. malariae*, and *P. knowlesi* [2]. Of these, *P. falciparum* is the most prevalent in Africa and is accountable for the majority of global malaria-related fatalities [1]. On the other hand, *P. vivax* dominates in regions such as the Americas, the eastern Mediterranean, and parts of Southeast Asia [3,4]. In 2021, reports from WHO estimated 619 million global malaria cases, resulting in approximately 568,000 deaths [1]. Despite extensive public health interventions over the past decade, the worldwide battle against malaria has encountered obstacles, including limited funding, rising drug and insecticide resistance, and challenges in accessing crucial malaria resources [5,6,7].

Oxidative stress is characterized by an imbalance between the accumulation of reactive oxygen species (ROS) and the body’s capability to neutralize them, leading to potential cellular harm [8,9]. The human body possesses an antioxidant defense mechanism that provides protection against oxidative harm instigated by these reactive oxidants [10]. This defense system significantly contributes to shielding cells against oxidative stress and injury. The main enzymatic antioxidants that serve as a vital line of protection against ROS include superoxide dismutase (SOD), catalase (CAT), glutathione peroxidase (GSH-Px), glutathione-S-transferase (GST), and peroxiredoxins [11]. The other lines of protection antioxidants are alpha-tocopherol (vitamin E), ascorbic acid (vitamin C), lipoic acid, selenium, copper, iron, zinc, glutathione (GSH), flavonoids, and carotenoids [12].

Carotenoids, which are naturally occurring pigmented compounds found in plants, algae, and certain bacteria and fungi, play a pivotal role in human nutrition due to their antioxidant properties. These compounds are known to quench singlet oxygen (^1^O_2_) and act as scavengers for ROS, with some even converting into vitamin A [13]. β-Carotene, which is notably prominent among carotenoids, is a primary colorant in human foods [14]. It is classified as an antioxidant based on its structural features and experimental findings, allowing it to counter lipid oxide and peroxide radicals effectively [15,16]. Furthermore, β-carotene neutralizes singlet molecular oxygen and addresses other ROS, mainly peroxyl radicals [14]. Beyond its antioxidant properties, β-carotene is also instrumental in immune functions [17]. Previous studies suggest that malaria infections trigger notable oxidative stress in hosts due to elevated ROS production, culminating in cellular damage and further complications [18,19].

Given carotenoids’ potent antioxidant nature, especially that of β-carotene, there is rising interest in exploring its potential protective role against the oxidative damage linked to malaria [20,21]. Despite the growing number of studies on this topic, individual results might be influenced by sample size, methodology, or demographic and geographic factors. By synthesizing the available evidence, this study aimed to offer a clearer picture of the relationship between β-carotene and malaria, highlight any consistencies or inconsistencies in the current research, and provide recommendations for future research directions. The findings from this study can be pivotal, not only for understanding the molecular mechanisms underpinning the interaction between β-carotene and malaria but also for population-based strategies aimed at mitigating the effects of the disease. Furthermore, by potentially uncovering the protective role of β-carotene, public health initiatives might be informed on dietary or supplemental interventions that could be explored in malaria-endemic regions.

## 2. Methods

### 2.1. Protocol

The systematic review and meta-analysis protocol were registered at PROSPERO (CRD42023453837). The systematic review and meta-analysis were developed in accordance with the Preferred Reporting Items for Systematic Reviews and Meta-Analyses (PRISMA) guidelines.

### 2.2. Search Strategy and Selection Criteria

A systematic search was conducted across various databases using a comprehensive combination of search terms: “(“beta Carotene” OR Betacarotene OR beta-Carotene OR Carotaben OR Max-Caro OR “Max Caro” OR MaxCaro OR Solatene OR Vetoron OR BellaCarotin OR Provatene OR Carotenoid OR Tetraterpenes OR Tetraterpene OR Carotenes OR Carotene) AND (malaria OR *Plasmodium* OR “*Plasmodium* Infection” OR “Remittent Fever” OR “Marsh Fever” OR Paludism)”. While the core strategy was retained, it was tailored slightly to suit the specific requirements of each database (details provided in Table S1). The databases explored included Nursing and Allied Health Premium, EMBASE, MEDLINE, Ovid, PubMed, and Scopus. The main objective of the search was to identify studies that explored the relationship between β-carotene levels and malaria. Additionally, to ensure comprehensiveness, Google Scholar was browsed, and reference lists of pertinent studies were meticulously reviewed.

### 2.3. Eligibility Criteria and Study Selection

Studies that investigated the relationship between β-carotene levels and malaria were focused on for inclusion. As an initial step in the study selection process, duplicate records were identified and removed. This left a subset of unique records that were then meticulously assessed. Titles and abstracts of these records were screened for relevance. Studies found to be unrelated to the target participant group or those that did not align with the intended outcomes were excluded at this juncture. Furthermore, studies characterized as reviews, letters, in vivo or in vitro studies, case reports, or conference abstracts were also excluded. The next step involved a detailed review of the full-text articles to confirm their eligibility. Specifically, any articles that did not provide data on β-carotene levels in malaria studies or did not meet other pertinent criteria were discarded. This selection process was diligently carried out by two independent authors, and in the event of any disagreements, a third author was consulted to reach a resolution.

### 2.4. Data Extraction and Quality Assessment

Information including the publication year, study design, geographical location, target species of *Plasmodium*, the target demographic, clinical status, and methods for malaria detection and β-carotene measurement were extracted from selected studies by two independent authors. Any disagreement was resolved through discussion to reach a consensus. A quality assessment of the selected studies was conducted to ensure methodological robustness using the JBI (Joanna Briggs Institute) checklists for case-control, cohort, and cross-sectional studies [22]. These checklists considered various criteria tailored to each study type, including exposure measurement, outcome assessment, confounding factors, and follow-up procedures.

### 2.5. Data Synthesis and Analysis

Qualitative synthesis was performed to provide insights into the key findings from the included studies. This synthesis summarized observations related to β-carotene levels in malaria patients compared with those without the disease. It also highlighted variations based on factors such as the disease severity and geographical location. A meta-analysis was conducted using data extracted from selected studies to determine potential differences in β-carotene levels between individuals diagnosed with malaria and uninfected controls. The effect size measure, namely, Hedges’s g, was chosen for its accuracy in dealing with studies of varying sizes and was presented with a 95% confidence interval [23]. A random-effects model using the DerSimonian and Laird method was employed [24], assuming that variations might exist in the underlying effects of the studies due to factors such as differing study populations or methodologies. The inconsistency index (I^2^) was utilized to measure the variability in the results from the individual studies [25]. I^2^ values of 25%, 50%, and 75% were interpreted as representing low, moderate, and high variability, respectively. Meta-regression and subgroup analyses were conducted to investigate potential sources of variability, such as the publication year, geographical location, age group of the participants, type of *Plasmodium* species, and method of β-carotene measurement. The robustness of the main meta-analysis findings was tested using a leave-one-out meta-analysis by re-running the meta-analysis, omitting one study at a time, and the influence of each study on the overall results was assessed [23]. Lastly, to detect potential publication bias, funnel plots and Egger’s test were employed, but this assessment was only considered reliable if at least 10 studies were included in the meta-analysis [26].

## 3. Results

### 3.1. Search Results

**Figure 1:**
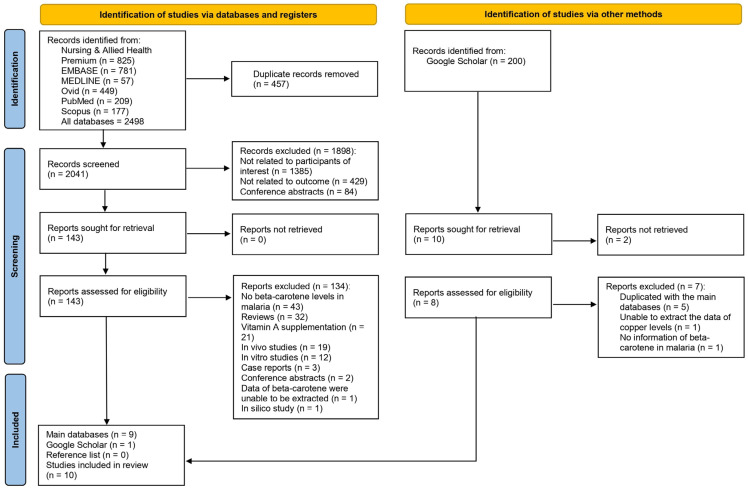
Study flow diagram showing the study selection processes.

Records were initially identified from several databases, yielding a total of 2498 entries. These databases included Nursing and Allied Health Premium with 825 records, EMBASE with 781, MEDLINE with 57, Ovid with 449, PubMed with 209, and Scopus with 177. Upon removing 457 duplicate records, 2041 records remained for screening. Of these, 1898 were subsequently excluded due to reasons such as not being related to the participants of interest (1385), not being pertinent to the outcome (429), or being conference abstracts (84). Consequently, 143 reports were retrieved and assessed for eligibility. However, 134 were excluded for various reasons including the absence of β-carotene levels in malaria studies and the nature of the study like in vivo or in vitro studies, among others. Finally, 10 studies were selected for the review [20,21,27,28,29,30,31,32,33,34], with 9 sourced from the main databases [20,21,27,28,29,30,31,32,33] and 1 from Google Scholar [34] (Figure 1).

### 3.2. Characteristics of Studies

Table 1 provides an overview of the characteristics of the 10 selected studies. The majority of these studies were published either before 2000 or between 2000 and 2009, with each period accounting for 40% of the total. In terms of design, half were case-control studies, 40% were cohort studies, and the remainder were cross-sectional. Geographically, most studies were conducted in Africa, especially Nigeria (50%), followed by Asia, with Thailand being the primary location (20%). As for the malaria-causing species, the dominant focus was on *P. falciparum* (60%). The majority of studies (60%) targeted children. When considering the clinical status, 70% of participants were symptomatic. The primary method for malaria detection was microscopy (presumably 90%, given the typographical error). β-Carotene measurements were mostly made using high-performance liquid chromatography (HPLC) in 60% of the studies, while 30% utilized other methods, and the remainder did not specify the method used. Details of all studies are presented in Table S2.

### 3.3. Quality of Studies

All case-control studies showed strong methodological quality, as indicated by their consistent positive responses to various criteria [27,28,32,33,34]. However, some studies [28,34] had gaps in addressing confounding factors, and all studies lacked clarity on the adequacy of the exposure period. All cohort studies uniformly measured exposures and reliably gauged outcomes. However, one study [31] was ambiguous in terms of handling confounding factors and follow-up procedures. One study [21] did not detail sufficient follow-up time. Benzecry et al. [29] fully adhered to all methodological criteria, whereas another study [20] overlooked confounding factors and did not address incomplete follow-ups. The cross-sectional study by Das et al. [30] underwent a methodological assessment based on eight criteria (Table S3).

### 3.4. Qualitative Synthesis

A significant portion of the included studies, including those by Adelekan et al. [27], Akpotuzor et al. [28], Das et al. [30], Farombi et al. [31], Thurnham D.I. and Singkamani R. (for urban areas) [33], and Uwah et al. [34], found that β-carotene levels were markedly reduced in patients with malaria when compared with those without the disease. Interestingly, Das et al. [30] further specified that among malaria patients, those with severe malaria had even more decreased β-carotene levels than those with non-severe forms of the disease. However, contrasting findings were reported by a few other studies. Specifically, Njoku et al. [20] and Stuetz et al. [32] observed no discernible difference in β-carotene levels either between malaria patients and uninfected controls or before and after treatment. This was also observed by Thurnham D.I. and Singkamani R. [33], but specifically in rural areas. Meanwhile, Benzecry et al. [29] reported a prevalence of β-carotene deficiency, but interestingly, found no linkage between this deficiency and higher incidence rates of malaria or any influence of a malaria episode on micronutrient levels. Nussenblatt et al. [21] offered a nuanced perspective, noting that among severely anemic malaria patients, β-carotene levels remained consistent pre- and post-treatment. In contrast, for patients without severe anemia, post-treatment β-carotene levels saw a significant uptick compared with the pre-treatment levels.

### 3.5. Meta-Analysis

**Figure 2:**
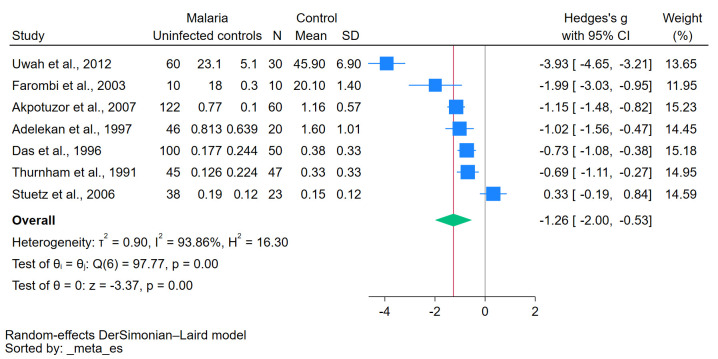
The pooled standardized mean difference in β-carotene levels between patients with malaria and uninfected controls. Symbols: blue box, effect estimate of each study; green diamond, pooled effect estimate; red line, pooled effect line; gray line, no effect line. References [24,25,27,28,29,30,31].

The meta-analysis results obtained using the data of seven studies investigated β-carotene levels in both malaria (421 patients) and uninfected controls (240 individuals) [24,25,27,28,29,30,31] show a significantly decreased β-carotene levels in malaria patients compared with the uninfected controls (*p* < 0.01, Hedges’s g: −1.26, 95% CI: −2.00–(−0.53), I^2^: 93.86%, seven studies, Figure 2). The results of individual studies show significantly decreased β-carotene levels in malaria patients compared with uninfected controls in six studies [27,28,30,31,33,34], but one study showed no difference in β-carotene levels between two groups [32].

The meta-regression analysis was further performed to test the influence of several factors on the pooled effect estimate. The meta-regression results show that the publication years significantly influenced the pooled β-carotene levels with a *p*-value of less than 0.01, explaining 58.88% of the variance and displaying high heterogeneity (I^2^ of 86.15%, Table 2). The continent was another significant covariate, accounting for 14.67% of the variance with a *p*-value of 0.022. Interestingly, methods for measuring β-carotene also showed significance, explaining 14.82% of the variance. However, the study design, age group, *Plasmodium* species, and clinical status did not significantly affect the pooled β-carotene levels, with each explaining no variance and showcasing high heterogeneity values.

**Figure 3:**
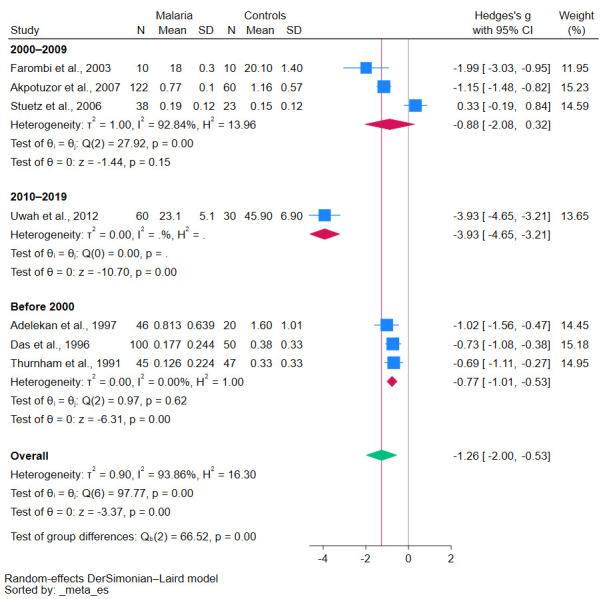
The pooled standardized mean difference in β-carotene levels between patients with malaria and uninfected controls stratified by publication year. Symbols: blue box, effect estimate of each study; green diamond, pooled effect estimate; red diamond, pooled effect estimate in the subgroup; red line, pooled effect line; gray line, no effect line. In the subgroup analysis for the years 2010–2019, there was no data available because only one study was included. References [24,25,27,28,29,30,31].

**Figure 4:**
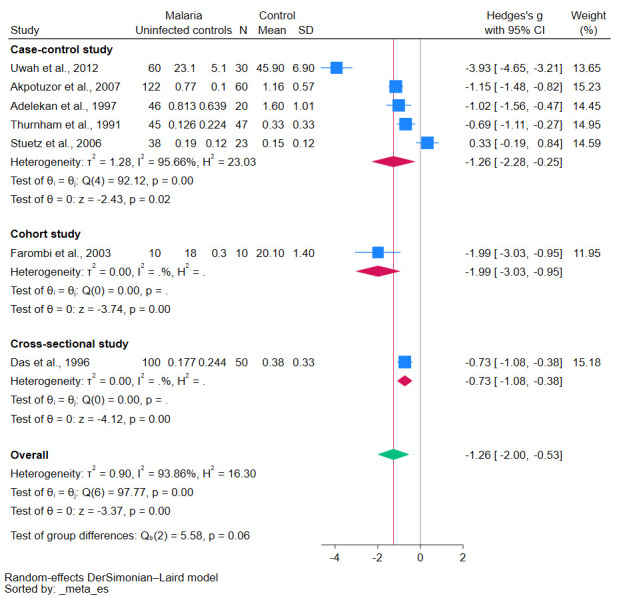
The pooled standardized mean difference in β-carotene levels between patients with malaria and uninfected controls stratified by study design. Symbols: blue box, effect estimate of each study; green diamond, pooled effect estimate; red diamond, pooled effect estimate in the subgroup; red line, pooled effect line; gray line, no effect line. In the subgroup analysis for the cohort study, there was no data available because only one study was included. References [24,25,27,28,29,30,31].

**Figure 5:**
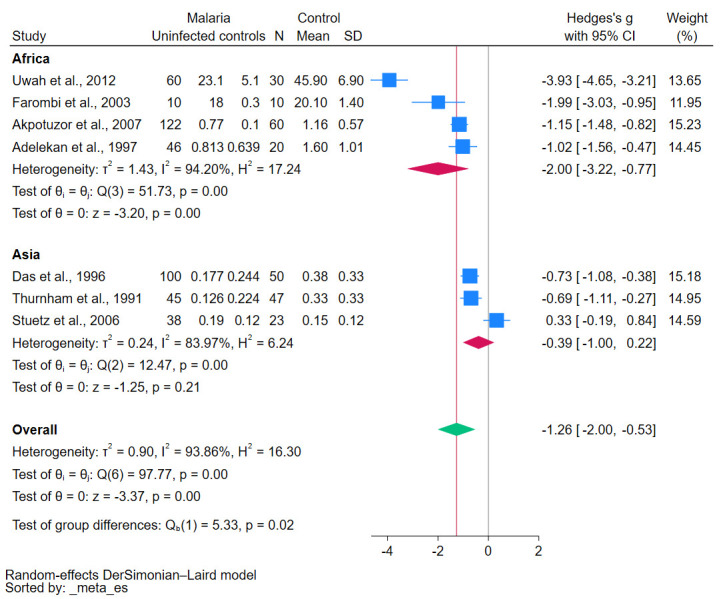
The pooled standardized mean difference in β-carotene levels between patients with malaria and uninfected controls stratified by continent. Symbols: blue box, effect estimate of each study; green diamond, pooled effect estimate; red diamond, pooled effect estimate in the subgroup; red line, pooled effect line; gray line, no effect line. References [24,25,27,28,29,30,31].

**Figure 6:**
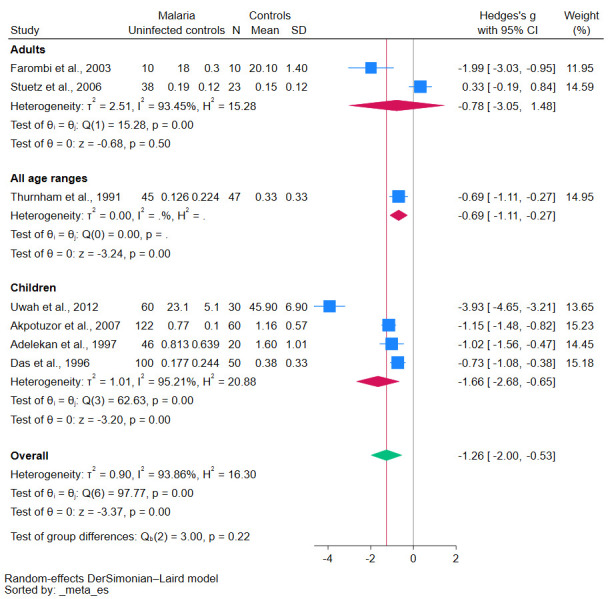
The pooled standardized mean difference in β-carotene levels between patients with malaria and uninfected controls stratified by age group. Symbols: blue box, effect estimate of each study; green diamond, pooled effect estimate; red diamond, pooled effect estimate in the subgroup; red line, pooled effect line; gray line, no effect line. In the subgroup analysis for all age ranges, there was no data available because only one study was included. References [24,25,27,28,29,30,31].

Subgroup analyses demonstrated various insights into the relationship between malaria and β-carotene levels. Studies conducted before 2010 revealed a significant decrease in β-carotene levels (*p*-value < 0.01, Figure 3). Similarly, case-control studies reinforced this trend, showing a statistically significant decrease in β-carotene levels (*p*-value = 0.02, Figure 4). Geographical analyses indicated that African studies consistently demonstrated this trend (*p*-value < 0.01, Figure 5). When data was stratified by age, the studies focusing specifically on children also corroborated these results, showing a significant decrease in β-carotene levels (*p*-value < 0.01, Figure 6).

**Figure 7:**
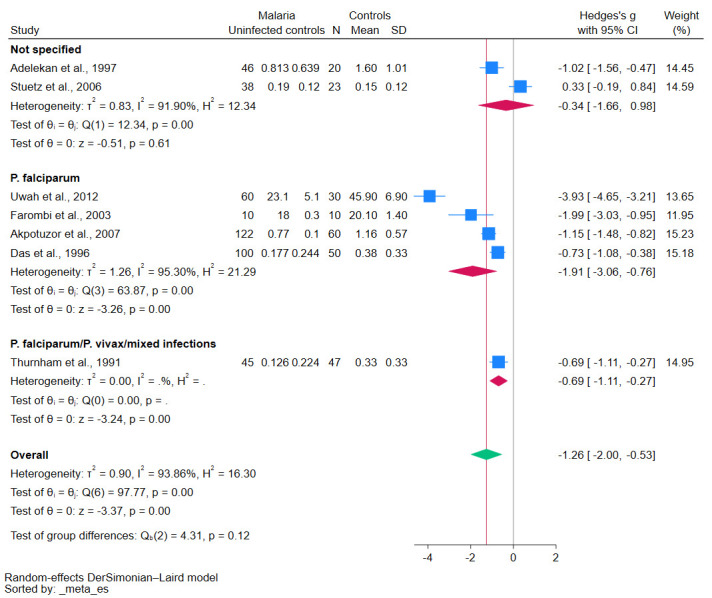
The pooled standardized mean difference in β-carotene levels between patients with malaria and uninfected controls stratified by *Plasmodium* species. Symbols: blue box, effect estimate of each study; green diamond, pooled effect estimate; red diamond, pooled effect estimate in the subgroup; red line, pooled effect line; gray line, no effect line. In the subgroup analysis for *P. falciparum*/*P. vivax*/mixed infections, there was no data available because only one study was included. References [24,25,27,28,29,30,31].

**Figure 8:**
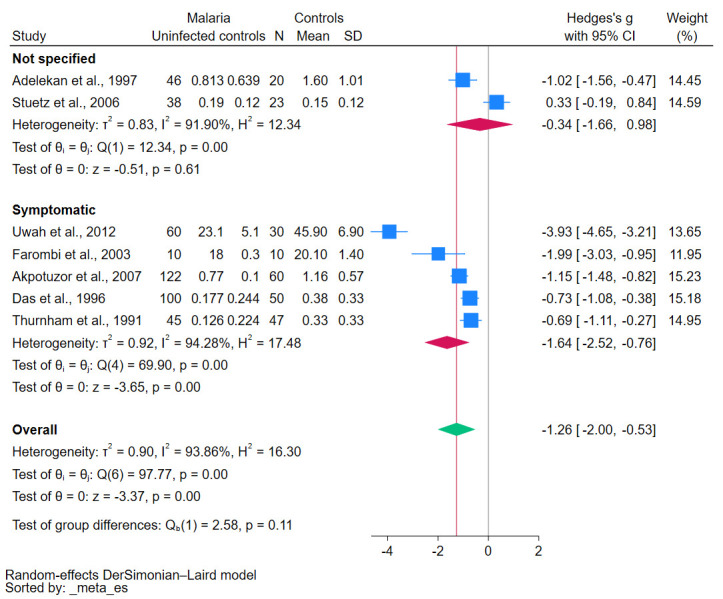
The pooled standardized mean difference in β-carotene levels between patients with malaria and uninfected controls stratified by clinical status. Symbols: blue box, effect estimate of each study; green diamond, pooled effect estimate; red diamond, pooled effect estimate in the subgroup; red line, pooled effect line; gray line, no effect line. References [24,25,27,28,29,30,31].

**Figure 9:**
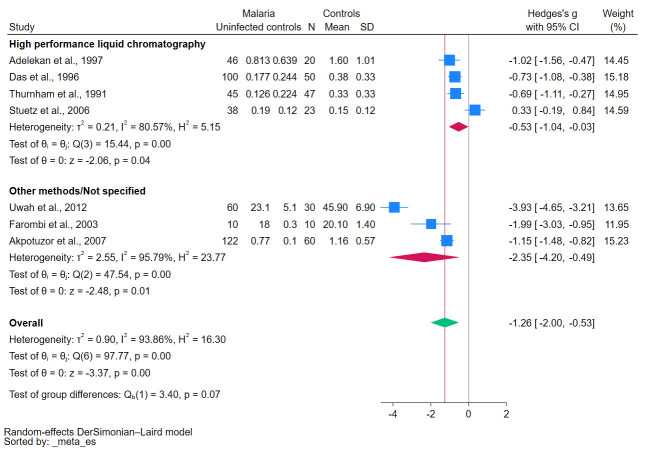
The pooled standardized mean difference in β-carotene levels between patients with malaria and uninfected controls stratified by the method for measuring β-carotene levels. Symbols: blue box, effect estimate of each study; green diamond, pooled effect estimate; red diamond, pooled effect estimate in the subgroup; red line, pooled effect line; gray line, no effect line. References [24,25,27,28,29,30,31].

Studies involving patients infected with *P. falciparum* reported significantly lower β-carotene levels (*p*-values < 0.01, Figure 7). Similarly, patients presenting with symp-tomatic malaria reported significantly lower β-carotene levels (*p*-values < 0.01, Figure 8). Notably, the methods used for the β-carotene measurements, such as HPLC versus other techniques, revealed significant variations in these levels (*p*-value < 0.05, Figure 9). The results of subgroup analyses that compared β-carotene levels in malaria patients to uninfected controls stratified by several factors are demonstrated in Table S4.

### 3.6. Sensitivity Analysis

**Figure 10:**
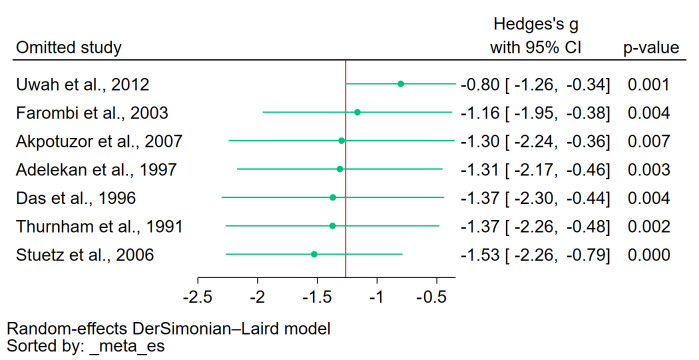
The leave-one-out meta-analysis results showing a significant decrease in β-carotene levels in patients with malaria compared with uninfected controls in each re-run analysis. Symbols: green dot, pooled effect estimate in each re-run analysis; red line, pooled effect line; gray line, no effect line. References [24,25,27,28,29,30,31].

The leave-one-out meta-analysis revealed significantly decreased β-carotene levels even though an individual study was removed in each re-run analysis (*p* < 0.05, Figure 10), suggesting that no single study disproportionately influenced the overall results and that the observed association between decreased β-carotene levels and malaria was consistent across various studies. The outcome confirmed the stability of the meta-analysis and reinforced the confidence in its conclusions.

### 3.7. Publication Bias

The publication bias was not performed because the studies included in the meta-analysis were less than 10 studies.

## 4. Discussion

The current systematic review and meta-analysis aimed to investigate the association between β-carotene levels and malaria. Comprehensive analyses were conducted using 10 included studies with varying characteristics and methodologies. A significant proportion of studies found that malaria patients had markedly reduced β-carotene levels compared with uninfected controls [27,28,30,31,33,34]. Furthermore, the meta-analysis conclusively demonstrated decreased β-carotene levels in malaria patients compared with uninfected controls. This is in alignment with previous studies that suggests that infections, including malaria, can affect micronutrient levels in the body [29,35,36,37]. β-Carotene, as a precursor to vitamin A, plays a crucial role in immune functions [14,17]. Reduced levels might contribute to a weakened immune response, making individuals more susceptible to infections. It is also possible that the life cycle of *Plasmodium* spp. and the consequent hemolysis could directly or indirectly affect β-carotene levels [38]. In addition, the increased utilization of provitamin A carotenoids during malaria infection might be another mechanism related to decreased β-carotene levels in malaria patients [21].

However, contrasting findings were also reported in some studies, as no discernible difference in β-carotene levels between malaria patients and uninfected controls was observed [20,32]. As the meta-analysis results conclusively demonstrated decreased β-carotene levels in malaria patients compared with uninfected controls with a high degree of statistical significance, several factors may affect the β-carotene levels during *Plasmodium* infection. The factors that may cause such disparities in results could arise from differences in study populations, geographical variations, dietary habits, or even methodological differences in β-carotene measurements. The differences in these factors were demonstrated in the results of the meta-regression analysis in which the publication year and the continent of the study were influential in determining the pooled β-carotene levels. It is noteworthy that the methods of measuring β-carotene also significantly influenced the results, underscoring the importance of standardized methodologies in research. The subgroup meta-analysis of studies conducted before 2000 showed a significant reduction in β-carotene levels without heterogeneity (I^2^ of 0%), indicating the reliability of the meta-analysis result. Nevertheless, the subgroup meta-analysis of studies conducted between 2000–2009 showed no significant result but with high heterogeneity (I^2^ of 92.84%), indicating the heterogenous meta-analysis result. The meta-regression indicated that the year of publication significantly influenced results, pointing to potential evolving methodologies, diagnostic tools, or other factors that might have changed over time, as the method for measuring β-carotene levels also affected the pooled effect estimate.

Beyond β-carotene, other dietary antioxidants and carotenoids are speculated to influence malaria’s susceptibility and severity. These dietary antioxidants include vitamins like alpha-tocopherol (vitamin E) and ascorbic acid (vitamin C), as well as other micronutrients, such as iron, selenium, copper, and zinc [11,12,39,40]. Furthermore, non-beta-carotene carotenoids, including lutein, zeaxanthin, and lycopene, possess antioxidant properties that might play a role in combating malaria parasites [30,41,42]. It is interesting to observe a significant difference in β-carotene levels between malaria patients and uninfected individuals in studies conducted in Africa, whereas no such difference was observed in Asian studies. One possible explanation is that the African population is more frequently exposed to *Plasmodium* infection compared with the Asian population. Faber et al. found that β-carotene-rich fruits and vegetables were rarely available in certain rural South African communities [43]. Consequently, increased exposure to *Plasmodium* infection might exacerbate micronutrient deficiencies, including that of β-carotene [29]. In addition, the subgroup analysis provided deeper insights, revealing that studies that focused on children or *P. falciparum* infections consistently highlighted the marked decrease in β-carotene levels among malaria patients. These results suggested that the African population, *P. falciparum*, and the age group of less than 18 years (children) were associated with a reduction in β-carotene in malaria patients. More importantly, the prominent decrease in β-carotene levels among symptomatic patients might indicate that the severity of the disease could be directly linked to micronutrient levels, which is a hypothesis that aligns with Das et al. [30], who observed more significant reductions in β-carotene levels among those with severe malaria. It is intriguing to note that while Benzecry et al. [29] reported β-carotene deficiency, they did not find a direct correlation with the incidence rates of malaria. Such observations highlight the intricate relationship between micronutrients and disease manifestations and underscore the importance of considering multiple confounding factors. The sensitivity analysis confirmed the robustness and stability of the results, affirming the consistency of the association between reduced β-carotene levels and malaria across various studies. While publication bias was not assessed due to the number of included studies being less than ten, it remains a critical aspect to consider in future systematic reviews and meta-analyses.

This study had limitations. First, the results of the meta-analysis exhibited a significant degree of heterogeneity, potentially complicating the ability to formulate overarching conclusions. Second, the included studies had different designs (case-control, cohort, and cross-sectional), which inherently come with their respective biases and limitations. Third, due to the number of studies being less than ten in the meta-analysis, the assessment for publication bias was not performed. It is commonly recommended that at least 10 studies be included to test for publication bias [26]. This limitation means that while the results of the meta-analysis provide valuable insights, there is potential, albeit unmeasured, for publication bias that might have affected the meta-analysis results.

Considering the association between malaria and decreased β-carotene levels, healthcare professionals in malaria-endemic regions should consider monitoring β-carotene and other vital micronutrients among patients diagnosed with malaria. Patients diagnosed with malaria, particularly those with severe symptoms, might benefit from β-carotene or vitamin A supplementation to potentially boost immune response and overall health. The benefits of β-carotene were corroborated by a prior study, which administered vitamin A and β-carotene supplements to reduce the risk of clinical malaria in children born to HIV-infected women [44]. To understand why malaria impacts β-carotene levels, studies could delve deeper into the molecular and biochemical mechanisms, exploring the life cycle of *Plasmodium* spp., hemolysis, and their interactions with micronutrients.

## 5. Conclusions

In conclusion, this systematic review and meta-analysis clarifies the potential association between β-carotene levels and malaria. However, it also highlights the need for more comprehensive studies, incorporating standardized methodologies and addressing potential confounding factors. Given the global burden of malaria, understanding such associations can have significant implications for public health strategies, nutritional interventions, and disease management.

## Figures and Tables

**Table 1:** Characteristics of studies.

Characteristics	No. (10 Studies)
**Publication year**	
Before 2000	4
2000–2009	4
2010–2019	2
**Study designs**	
Case-control studies	5
Cohort studies	4
Cross-sectional studies	1
**Study areas**	
**Africa**	
Nigeria	5
Uganda	1
**Asia**	
Thailand	2
India	1
**South America**	
Brazil	1
***Plasmodium* species**	
*P. falciparum*	6
*P. vivax*	1
*P. falciparum*, *P. vivax*, mixed infections	1
Not specified	2
**Age group**	
Children	6
Adults	2
Children, adults	2
**Clinical status**	
Symptomatic	7
Not specified	3
**Methods for malaria detection**	
Microscopy	9
Microscopy/PCR	1
**Methods for β-carotene measurement**	
HPLC	6
Other methods	3
Not specified	1

HPLC, high-performance liquid chromatography; PCR, polymerase chain reaction.

**Table 2:** Meta-regression results.

Meta-Analysis of β-Carotene	Covariates	*p*-Value	tau^2^	I^2^ (%)	R-Squared (%)	Number of Studies
Malaria patients vs. uninfected individuals	Publication years	<0.01	0.368	86.15	58.88	7
	Study design	0.760	1.284	95.66	0.00	7
	Continent	0.022	0.764	92.21	14.67	7
	Age group	0.169	0.885	94.42	1.14	7
	*Plasmodium* species	0.231	1.167	94.75	0.00	7
	Clinical status	0.117	0.902	93.92	0.00	7
	Methods for β-carotene	0.011	0.763	92.06	14.82	7

Not assessed because of collinearity.

## Data Availability

All data relating to the present study are available in this manuscript and supplementary files.

## Supplementary Materials

The following supporting information can be downloaded at: https://www.mdpi.com/article/10.3390/antiox12091687/s1, Table S1: Search terms; Table S2: Details of the included studies; Table S3: Quality of included studies; Table S4: Subgroup analyses of β-carotene levels between malaria cases and uninfected controls.
