# Supplementary material for: The Association between Malaria and β-Carotene Levels: A Systematic Review and Meta-Analysis
Source: Antioxidants (Basel). 2023 Aug 29;12(9):1687. doi: 10.3390/antiox12091687 (PMC10525879; doi:10.3390/antiox12091687)
Supplement: Supplementary file 1 [file antioxidants-12-01687-s001.zip › Table S1. Search terms.pdf]

The Association between Malaria and β-Carotene Levels: A Systematic Review and Meta-Analysis

Kwuntida Uthaisar Kotepui[1†], Aongart Mahittikorn[2†], Polrat Wilairatana[3*], Frederick Ramirez Masangkay[4], Manas Kotepui[1]*

[1]Medical Technology, School of Allied Health Sciences, Walailak University, Thasala, Nakhon Si Thammarat 80160, Thailand

[2]Department of Protozoology, Faculty of Tropical Medicine, Mahidol University, Bangkok 10400, Thailand

[3]Department of Clinical Tropical Medicine, Faculty of Tropical Medicine, Mahidol University, Bangkok 10400, Thailand

[4]Department of Medical Technology, Faculty of Pharmacy, University of Santo Tomas, Manila 1000, Philippines

*Corresponding author

† These authors contributed equally to this work.

Kwuntida Uthaisar Kotepui: kwuntida.ut@wu.ac.th

Aongart Mahittikorn: aongart.mah@mahidol.ac.th

Frederick Ramirez Masangkay: frederick_masangkay2002@yahoo.com

Polrat Wilairatana: polrat.mah@mahidol.ac.th

Manas Kotepui manas.ko@wu.ac.th, Tel+ :.66954392469

Table S1. Search terms

General keywords

(“beta Carotene” OR Betacarotene OR beta-Carotene OR Carotaben OR Max-Caro OR “Max Caro” OR MaxCaro OR Solatene OR Vetoron OR BellaCarotin OR Provatene OR Carotenoid OR Tetraterpenes OR Tetraterpene OR Carotenes OR Carotene) AND (malaria

OR plasmodium OR “Plasmodium Infection“ OR “Remittent Fever“ OR “Marsh Fever“ OR Paludism)

PubMed 11 August 2023

No.	Key concept	Search terms	Results
1.	beta Carotene	(“beta Carotene”[Text Word] OR “beta Carotene”[MeSH Terms] OR Betacarotene[Text Word] OR Betacarotene[MeSH Terms] OR beta-Carotene[Text Word] OR beta-Carotene[MeSH Terms] OR Carotaben[Text Word] OR Max-Caro[Text Word] OR “Max Caro”[Text Word] OR MaxCaro[Text Word] OR Solatene[Text Word] OR Vetoron[Text Word] OR BellaCarotin[Text Word] OR Provatene[Text Word] OR Carotenoid[MeSH Terms] OR Carotenoid[Text Word] OR Tetraterpenes OR Tetraterpene[Text Word] OR Carotenes[Text Word] OR Carotene[Text Word])	112,384
2.	Malaria	malaria[Text Word] OR plasmodium[Text Word] OR malaria[MeSH Terms] OR “Remittent Fever“[Text Word] OR “Marsh Fever“[Text Word] OR Paludism[Text Word]	119,033
3.	1 AND 2	(“beta Carotene”[Text Word] OR “beta Carotene”[MeSH Terms] OR Betacarotene[Text Word] OR Betacarotene[MeSH Terms] OR beta-Carotene[Text Word] OR beta-Carotene[MeSH Terms] OR Carotaben[Text Word] OR Max-Caro[Text Word] OR “Max Caro”[Text Word] OR MaxCaro[Text Word] OR Solatene[Text Word] OR Vetoron[Text Word] OR BellaCarotin[Text Word] OR Provatene[Text Word] OR Carotenoid[MeSH Terms] OR Carotenoid[Text Word] OR Tetraterpenes OR Tetraterpene[Text Word] OR Carotenes[Text Word] OR Carotene[Text Word]) AND (malaria[Text Word] OR plasmodium[Text Word] OR malaria[MeSH Terms] OR “Remittent Fever“[Text Word] OR “Marsh Fever“[Text Word] OR Paludism[Text Word])	209

Embase 11 August 2023

No.	Key concept	Search terms	Results
1.	beta Carotene	(“beta Carotene”:ti,ab,kw,de OR “beta Carotene”/exp OR Betacarotene:ti,ab,kw,de OR Betacarotene/exp OR beta-Carotene:ti,ab,kw,de OR beta-Carotene/exp OR Carotaben:ti,ab,kw,de OR Max-Caro:ti,ab,kw,de OR “Max Caro”:ti,ab,kw,de OR MaxCaro:ti,ab,kw,de OR Solatene:ti,ab,kw,de OR Vetoron:ti,ab,kw,de OR	191,207

		BellaCarotin:ti,ab,kw,de OR Provatene:ti,ab,kw,de OR Carotenoid/exp OR Carotenoid:ti,ab,kw,de OR Tetraterpenes:ti,ab,kw,de OR Tetraterpene:ti,ab,kw,de OR Carotenes:ti,ab,kw,de OR Carotene:ti,ab,kw,de)	
2.	Malaria	malaria:ti,ab,kw,de OR plasmodium:ti,ab,kw,de OR ‘Remittent Fever’:ti,ab,kw,de OR ‘Marsh Fever’:ti,ab,kw,de OR Paludism:ti,ab,kw,de OR malaria/exp	157,159
3.	1 AND 2	(“beta Carotene”:ti,ab,kw,de OR “beta Carotene”/exp OR Betacarotene:ti,ab,kw,de OR Betacarotene/exp OR beta-Carotene:ti,ab,kw,de OR beta-Carotene/exp OR Carotaben:ti,ab,kw,de OR Max-Caro:ti,ab,kw,de OR “Max Caro”:ti,ab,kw,de OR MaxCaro:ti,ab,kw,de OR Solatene:ti,ab,kw,de OR Vetoron:ti,ab,kw,de OR BellaCarotin:ti,ab,kw,de OR Provatene:ti,ab,kw,de OR Carotenoid/exp OR Carotenoid:ti,ab,kw,de OR Tetraterpenes:ti,ab,kw,de OR Tetraterpene:ti,ab,kw,de OR Carotenes:ti,ab,kw,de OR Carotene:ti,ab,kw,de) AND (malaria:ti,ab,kw,de OR plasmodium:ti,ab,kw,de OR ‘Remittent Fever’:ti,ab,kw,de OR ‘Marsh Fever’:ti,ab,kw,de OR Paludism:ti,ab,kw,de OR malaria/exp)	711

Scopus 11 August 2023

No.	Key concept	Search terms	Results
1.	beta Carotene	TITLE-ABS-KEY (“beta Carotene” OR Betacarotene OR beta-Carotene OR Carotaben OR Max-Caro OR “Max Caro” OR MaxCaro OR Solatene OR Vetoron OR BellaCarotin OR Provatene OR Carotenoid OR Tetraterpenes OR Tetraterpene OR Carotenes OR Carotene)	100,834
2.	Malaria	TITLE-ABS-KEY (malaria OR plasmodium OR “Plasmodium Infection“ OR “Remittent Fever“ OR “Marsh Fever“ OR Paludism)	158,011
3.	1 AND 2	(TITLE-ABS-KEY ("beta Carotene" OR betacarotene OR beta-carotene OR carotaben OR max-caro OR "Max Caro" OR maxcaro OR solatene OR vetoron OR bellacarotin OR provatene OR carotenoid OR tetraterpenes OR tetraterpene OR carotenes OR carotene)) AND (TITLE-ABS-KEY (malaria OR plasmodium OR "Plasmodium Infection" OR "Remittent Fever" OR "Marsh Fever" OR paludism))	177

MEDLINE 10 August 2023

No.	Key concept	Search terms	Results

1.	beta Carotene AND Malaria	(“beta Carotene” OR Betacarotene OR beta-Carotene OR Carotaben OR Max-Caro OR “Max Caro” OR MaxCaro OR Solatene OR Vetoron OR BellaCarotin OR Provatene) AND (malaria OR plasmodium OR “Plasmodium Infection“ OR “Remittent Fever“ OR “Marsh Fever“ OR Paludism)	57

Ovid 10 August 2023

No.	Key concept	Search terms	Results
1.	beta Carotene AND Malaria	(“beta Carotene” OR Betacarotene OR beta-Carotene OR Carotaben OR Max-Caro OR “Max Caro” OR MaxCaro OR Solatene OR Vetoron OR BellaCarotin OR Provatene OR Carotenoid OR Tetraterpenes OR Tetraterpene OR Carotenes OR Carotene) AND (malaria OR plasmodium OR “Plasmodium Infection“ OR “Remittent Fever“ OR “Marsh Fever“ OR Paludism) {Including Limited Related Terms}	449

Nursing & Allied Health Premium 10 August 2023

No.	Key concept	Search terms	Results
1.	beta Carotene AND Malaria	(“beta Carotene” OR Betacarotene OR beta-Carotene OR Carotaben OR Max-Caro OR “Max Caro” OR MaxCaro OR Solatene OR Vetoron OR BellaCarotin OR Provatene OR Carotenoid OR Tetraterpenes OR Tetraterpene OR Carotenes OR Carotene) AND (malaria OR plasmodium OR “Plasmodium Infection“ OR “Remittent Fever“ OR “Marsh Fever“ OR Paludism)	

Google Scholar 10 August 2023

No.	Key concept	Search terms	Results
1.	beta Carotene AND Malaria	“beta Carotene” AND malaria	200
