# Supplementary material for: The Association between Malaria and β-Carotene Levels: A Systematic Review and Meta-Analysis
Source: Antioxidants (Basel). 2023 Aug 29;12(9):1687. doi: 10.3390/antiox12091687 (PMC10525879; doi:10.3390/antiox12091687)
Supplement: Supplementary file 1 [file antioxidants-12-01687-s001.zip › Table S4. Subgroup analysis.pdf]

The Association between Malaria and β-Carotene Levels: A Systematic Review and Meta-Analysis

Kwuntida Uthaisar Kotepui[1†], Aongart Mahittikorn[2†], Polrat Wilairatana[3*], Frederick Ramirez Masangkay[4], Manas Kotepui[1]*

[1]Medical Technology, School of Allied Health Sciences, Walailak University, Thasala, Nakhon Si Thammarat 80160, Thailand

[2]Department of Protozoology, Faculty of Tropical Medicine, Mahidol University, Bangkok 10400, Thailand

[3]Department of Clinical Tropical Medicine, Faculty of Tropical Medicine, Mahidol University, Bangkok 10400, Thailand

[4]Department of Medical Technology, Faculty of Pharmacy, University of Santo Tomas, Manila 1000, Philippines

*Corresponding author

† These authors contributed equally to this work.

Kwuntida Uthaisar Kotepui: kwuntida.ut@wu.ac.th

Aongart Mahittikorn: aongart.mah@mahidol.ac.th

Frederick Ramirez Masangkay: frederick_masangkay2002@yahoo.com

Polrat Wilairatana: polrat.mah@mahidol.ac.th

Manas Kotepui manas.ko@wu.ac.th, Tel+ :.66954392469

Table S4. Subgroup analyses of β-carotene levels between malaria cases and uninfected controls.

Subgroup analyses	*P* value	Hedges' g (95% CI)	I^2	Number of studies
**Publication years**				
Before 2010	< 0.01	-0.77 (-1.01–(-0.53))	0	3
2010-2019	N/A	-3.93 (-4.65–(-3.21))	N/A	1
2010-2023	0.15	-0.88 (-2.08–0.32)	92.84	3
**Study design**				
Case-control study	0.02	-1.26 (-2.28–(-0.25))	95.66	5
Cohort study	N/A	-1.99 (-3.03–(-0.95))	N/A	1
Cross-sectional study	N/A	-0.73 (-1.08–(-0.38))	N/A	1
**Continent**				
Africa	< 0.01	-2.00 (-3.22–(-0.77))	94.20	4
Asia	0.21	-0.39 (-1.00–0.22)	83.97	3
**Participant's groups**				
Children	< 0.01	-1.66 (-2.68–(-0.65))	95.21	4
Adults	N/A	-1.99 (-3.03–(-0.95))	N/A	1
Children, adults	0.71	-0.19 (-1.19–0.81)	89.02	1
***Plasmodium* species**				
P. falciparum	< 0.01	-1.91 (-3.06–(-0.76))	95.30	4
P. falciparum, P. vivax, mixed infections	N/A	-0.69 (-1.11–(-0.27))	N/A	1
Not specified	0.61	-0.34 (-1.66–0.98)	91.90	2
**Clinical status**				
Symptomatic	< 0.01	-1.64 (-2.52–(-0.76))	94.28	5
Not specified	0.61	-0.34 (-1.66–0.98)	91.90	2
**Methods for β-carotene**				
HPLC	0.04	-0.53 (-1.04–(-0.03))	80.57	4
Other methods/Not specified	0.01	-2.35 (-4.20–(-0.49))	95.79	3

Abbreviations: CI, confidence interval; HPLC, High performance liquid chromatography; N/A, not assessed
